# Distinct Response of Circulating microRNAs to the Treatment of Pancreatic Cancer Xenografts with FGFR and ALK Kinase Inhibitors

**DOI:** 10.3390/cancers14061517

**Published:** 2022-03-16

**Authors:** Ivana Peran, Eveline E. Vietsch, Gai Yan, Anna T. Riegel, Anton Wellstein

**Affiliations:** Georgetown-Lombardi Comprehensive Cancer Center, Department of Oncology, Georgetown University Medical Center, Washington, DC 20057, USA; ip62@georgetown.edu (I.P.); e.vietsch@erasmusmc.nl (E.E.V.); gy63@georgetown.edu (G.Y.); ariege01@georgetown.edu (A.T.R.)

**Keywords:** microRNA, miR, circulating miR, pancreatic cancer, pancreatic adenocarcinoma, treatment response, biomarker, stroma

## Abstract

**Simple Summary:**

Pancreatic cancer is among the top three leading causes of cancer-related death in the United States, mainly due to late diagnosis after the disease has already spread to other organs. Hence, timely and efficient treatments are necessary to improve outcomes. In this study, we explored the use of circulating microRNAs (miRs) collected from the peripheral blood of pancreatic cancer-bearing mice for assessment of treatment efficacy early in the treatment schedule. MicroRNA changes in serum samples precede histologic changes during the treatments, suggesting the use of circulating microRNA as early indicators of response to treatment. These easily-accessible biomarkers can be sampled repeatedly and could guide timely treatment decisions.

**Abstract:**

Pancreatic adenocarcinoma is typically detected at a late stage and thus shows only limited sensitivity to treatment, making it one of the deadliest malignancies. In this study, we evaluate changes in microRNA (miR) patterns in peripheral blood as a potential readout of treatment responses of pancreatic cancer to inhibitors that target tumor–stroma interactions. Mice with pancreatic cancer cell (COLO357PL) xenografts were treated with inhibitors of either fibroblast growth factor receptor kinase (FGFR; PD173074) or anaplastic lymphoma kinase receptor (ALK; TAE684). While both treatments inhibited tumor angiogenesis, signal transduction, and mitogenesis to a similar extent, they resulted in distinct changes in circulating miR signatures. Comparison of the miR pattern in the tumor versus that in circulation showed that the inhibitors can be distinguished by their differential impact on tumor-derived miRs as well as host-derived circulating miRs. Distinct signatures that include circulating miR-1 and miR-22 are associated with the efficacy of ALK and FGFR inhibition, respectively. We propose that monitoring changes in circulating miR profiles can provide an early signature of treatment response or resistance to pathway-targeted drugs, and thus provide a non-invasive measurement to rapidly assess the efficacy of candidate therapies.

## 1. Introduction

Pancreatic ductal adenocarcinoma (PDAC) has one of the lowest overall five-year survival rates of any cancer at ~10%, due mainly to the lack of symptoms during early disease and thus its detection only at late stages [1]. This has prompted research on new approaches to diagnose early-stage pancreatic cancer as well as to develop new therapeutic strategies for progressed disease. While the majority of research on cancer treatment focuses on targeting the epithelial component of cancer, this approach has not been very effective in PDAC, a desmoplastic disease in which stroma is a major component of the tumor (up to 80%) and thought to contribute to drug resistance [1,2,3]. Thus, disruption of tumor–stroma interactions may be useful in treating PDAC. Considering the dual role of cancer-associated fibroblasts in PDAC [4,5,6,7,8], rather than deplete stroma from the tumor microenvironment; we propose to affect the cross-talk between cancer cells, CAFs, endothelial cells, immune cells, and other stromal components by altering signaling that is involved in perpetuating tumor growth, similarly to other recently published preclinical studies [9]. Here, we evaluated two tyrosine kinase inhibitors, PD173074, which inhibits FGFR1 and 3, and TAE684, which is a selective inhibitor of the ALK receptor kinase [10,11,12]. Both receptor kinases and their ligands are associated with pancreatic cancer (Supplementary Figure S1) [13,14,15]. In addition to being increased in pancreatic cancer tissues relative to normal pancreas, FGF signaling is crucial for tumor–stroma crosstalk and angiogenesis [2,16]. Similarly, the ALK ligand pleiotrophin (PTN) is elevated in serum and tissues from pancreatic cancer patients [17], and it has been shown that PTN levels are associated with the malignant progression of human pancreatic cancer, demonstrating the clinical significance of this signaling pathway [14]. FGFR-dependent signaling is essential for pancreatic cancer growth, and is involved in the proliferation (specifically FGFR1-IIIb), adhesion, and movement of pancreatic ductal cells [18,19]. Furthermore, it has been shown that FGFR signaling plays a role in pancreatic cancer cells stemness and can be therapeutically targeted by PD173074, dovitinib, and AZD4547 [20,21]. Additionally, it has been shown that blocking ALK signaling by TAE684 exerts anti-tumor effects in pancreatic cancer [22], while ALK inhibition by crizotinib suppresses pancreatic tumor growth, cell proliferation, and angiogenesis and induces apoptosis [23]. Finally, clinical trials (NCT02227940, NCT01497392) using ALK and FGFR inhibitors have been approved for advanced pancreatic cancer, further providing a rationale for testing the above-mentioned inhibitors in preclinical mouse models of pancreatic cancer.

Treatment responses of the host and/or the tumor are likely to be reflected in specific changes of circulating biomarkers that can be assessed from analyses of serial blood draws and be translatable to patient monitoring. Here, we focused on changes in levels of circulating microRNAs (miRs) to provide a sufficiently complex readout that reflects drug-induced pathway alterations. In the decade following their discovery, the association of miRs with cancer has been extensively studied [24,25]. Importantly, certain miRs can accurately identify cancer tissue of origin, and identification of miRs in the circulation has led to the possibility of using circulating miRs as diagnostic biomarkers for cancer detection [26,27,28]. An extensive study published in 2011 demonstrated the potential use of circulating miRs as biomarkers for different disease conditions, including cancer, as miR profiles successfully distinguished different diseases from each other and from healthy controls [29]. Interestingly, circulating miRs may have endocrine and/or paracrine functions, suggesting that miRs shed into circulation might have either oncogenic or tumor suppressing activities by signaling distant recipient cells, tissues, or organs [30,31,32]. Finally, detection of circulating miRs is feasible over a wide dynamic range thanks to PCR-based detection, and is expandable to different sets of miRs by selecting appropriate primer sets.

We hypothesized that the expression of circulating miRs may be altered as a result of targeted therapy and that the altered expression profile may be used to predict treatment response. To test this hypothesis, we evaluated tumor and serum miR expression immediately after short-term treatment of pancreatic tumor xenografts with either the FGFR or ALK inhibitor in a COLO357PL [33,34,35] model expressing both FGFR and ALK receptors [20,22]. Both kinase inhibitors had similar inhibitory effects on mitogenesis and angiogenesis. However, we found a distinct pattern of serum miRs that was associated with each kinase inhibitor after a short seven-day treatment. Additionally, we used the FGFR inhibitor in another mouse model of cancer to assess the inhibitor-specific miR pattern upon short-term treatment. For that study, we selected the breast cancer MDA-MB-231 xenograft model that contains *KRAS* mutation [36], which is also present in 80–95% of pancreatic cancer specimen from patients [37]. Furthermore, it has been shown that FGFR signaling plays an important role in breast cancer cells themselves, as well as other cell types that contribute to the breast cancer microenvironment such as fibroblasts, endothelial cells, immune cells, and others (Supplementary Figure S1) [38]. Finally, we assessed to what extent the drug effect on tumor tissues can be distinguished from drug effects on the host based on the comparison of miR levels in the tumor tissues and the serum. From our data, we suggest that the distinct changes in serum miR profile after the short treatment can predict drug efficacy observed after a longer treatment period.

## 2. Materials and Methods

### 2.1. Cell Culture

Human pancreatic cancer cells COLO357PL [34], obtained from Dr. Isaiah Fidler (University of Texas, M. D. Anderson Cancer Center, Houston, TX, USA), and human breast cancer cells, MDA-MB-231, obtained from the Tissue Culture Shared Resource at Georgetown University, Washington, DC, USA were grown in Dulbecco’s Modified Eagle Medium (DMEM; Invitrogen, Waltham, MA, USA) with the addition of 10% fetal bovine serum (FBS; Peak Serum, Inc., Wellington, CO, USA). To obtain cell proliferation rate and the rate of pancreatic cancer cell disruption of endothelial cell monolayer, we used the electric cell–substrate impedance sensing system. In a proliferation assay, 8000 cells per well were plated in duplicates in xCELLigence E-plate array (Roche Applied Biosciences, Indianapolis, IN, USA) and impedance was measured until confluency was reached. Cells were plated in DMEM + 10% FBS with addition of dimethyl sulfoxide (DMSO; Fisher Scientific, Waltham, MA, USA) as a control or with different concentrations of PD173074 (6 nM, 25 nM and 100 nM) or TAE684 drug (2 nM, 8 nM and 32 nM). In the endothelial cell invasion assay, 30000 human umbilical vein endothelial cells (HUVEC, obtained from Cambrex Biosciences, Walkersville, MD, USA) per well were plated in duplicates in EGM-2 media (EBM-2 medium with supplements and growth factors; Lonza, Walkersville, MD, USA). Approximately 24 h later, when HUVEC cells formed a monolayer, 6000 pretreated COLO357PL cells per well were added in DMEM + 10% FBS in addition of DMSO as a vehicle control or 100 nM PD173074 and 32 nM TAE684. Disruption of endothelial monolayer was followed during the next 6 h.

### 2.2. Treatment Compounds

PD173074 (Calbiochem, MilliporeSigma, Burlington, MA, USA) and TAE684 (Selleck Chemicals, Houston, TX, USA) were initially dissolved in DMSO at 10 mg/mL, aliquoted, and stored at −20 °C while protected from light. For in vitro studies, both drugs were diluted in 1× PBS (Invitrogen, Waltham, MA, USA), whereas for in vivo studies TAE684 was diluted in peanut oil (Spectrum Chemical Mfg. Corp., New Brunswick, NJ, USA).

### 2.3. Xenograft Mouse Model

All studies performed in mice received ethical approval by the Georgetown University Animal Care and Use Committee under animal protocol #11-032. One million COLO357PL cells were injected subcutaneously bilaterally into athymic nude mice. Tumors were measured daily, and after they reached measurable size (≥25 mm^2^) 7 days of treatment with PD173074 or TAE684 was initiated. Six to seven mice per experimental treatment group were used. The PD173074 drug was administered daily intraperitoneally (1 mg/kg) as established previously for in vivo studies [39]. TAE684 was administered daily by oral gavage at 10 mg/kg. The control group was injected daily intraperitoneally with DMSO diluted in saline.

In a separate xenograft model, 0.75 million MDA-MB-231 cells were injected subcutaneously bilaterally into nude mice. Two days later an 11-day treatment of mice with PD173074 was initiated. The dose and drug administration were the same as in the pancreatic cancer xenograft mouse model described above. Each treatment group consisted of 3–4 mice.

After seven days of treatment in the pancreatic cancer model and eleven days in the breast cancer model, mice were euthanized and blood samples and tumors were collected. Blood samples were collected in serum gel tubes with clotting gel activator (#41.1500.005, Sarstedt, Newton, NC, USA) and centrifuged at 10,000× *g* for 5 min. Serum was immediately stored at −80 °C.

### 2.4. Immunohistochemistry

Formalin-fixed and paraffin-embedded (FFPE) tumor tissue sections were stained with hematoxylin and eosin (H&E), von Willebrand factor (vWF) (#AB7356, Millipore, Burlington, MA, USA) phospho-FGFR1 (#PAB16969, Abnova, Walnut, CA, USA), and BrdU (Invitrogen, Life Technologies, Carlsbad, CA, USA). TUNEL staining (terminal deoxynucleotidyl transferase dUTP nick end labeling) was used to detect DNA fragmentation generated during apoptosis (#S7100, ApopTag Peroxidase In Situ Apoptosis Detection Kit, Millipore, Burlington, MA, USA). 

### 2.5. miR Analysis

Equal volumes of serum samples from mice belonging to the same treatment group were pooled together, followed by miR isolation using a miRNeasy Mini Kit (Qiagen, Germantown, MD, USA) with an RNeasy MiniElute Cleanup Kit (Qiagen). miR was reverse transcribed to cDNA using an RT^2^ miR First Strand Kit (SABiosciences, Germantown, MD, USA) on an Eppendorf Thermal Cycler (Eppendorf, Farmingham, MA, USA), according to the manufacturer’s protocol. A human genome wide miR array (RT^2^ miRNA PCR Array: Human Genome V2.0 (Set 1), SABiosciences, Germantown, MD, USA), or mouse cancer miR array (SABiosciences, Germantown, MD, USA) based on quantitative polymerase chain reaction (qPCR) was performed on an iCycler (BioRad, Hercules, CA, USA) using RT^2^ SYBR Green/Fluorescein qPCR master mix (SABiosciences, Germantown, MD, USA) according to the protocol. The top hit miRs were confirmed by running separate qPCR reactions. Furthermore, serum versus tumor miR expression levels were analyzed.

### 2.6. BrdU Labeling for Proliferating Cells

Four hours before euthanasia and collecting tissues, mice were injected intraperitoneally with BrdU solution in concentration of 5 mg/mL (Invitrogen, Life Technologies, Carlsbad, CA, USA). The volume injected in μL was 20× the body weight in grams. FFPE tumor tissues were stained using the manufacturer’s protocol (Invitrogen, Life Technologies, Carlsbad, CA, USA). 

### 2.7. Statistical Analysis

Data were analyzed with software available on the SABiosciences website, GraphPad Prism (GraphPad Software, La Jolla, CA, USA), and Ingenuity Pathway Analysis (IPA) software (Ingenuity^®^ Systems, Redwood City, CA, USA); *p* < 0.05 was considered as significant.

## 3. Results

### 3.1. Inhibition of FGFR and ALK Kinase Activity Reduces Invasiveness of Pancreatic Cancer Cells In Vitro

**Figure 1:**
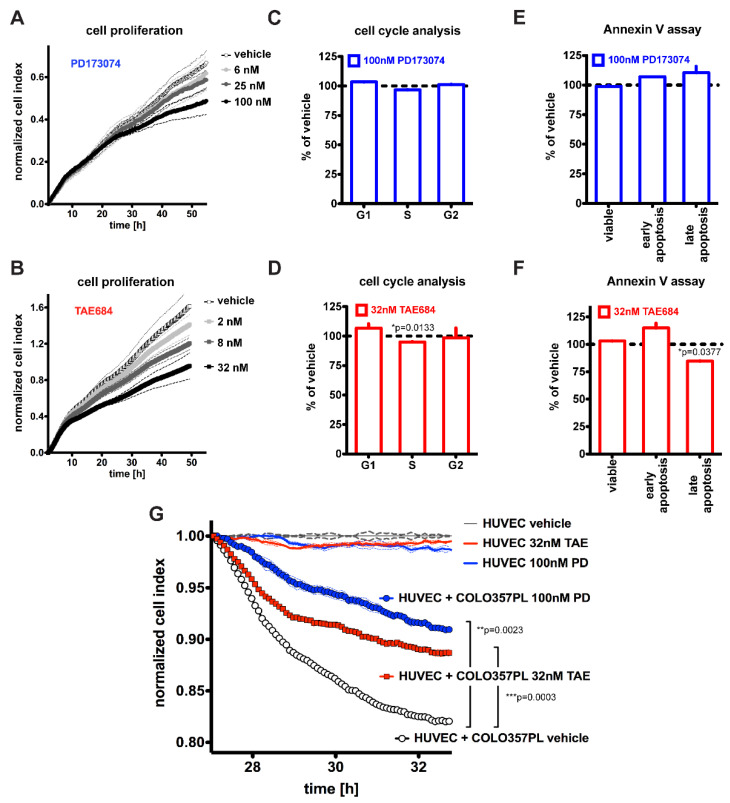
Response of COLO357PL cells to PD173074 or TAE684 in vitro. (**A**,**B**): Proliferation assays. COLO357PL cells were plated in the presence of drug in duplicates and measurements were made every 15 min. Cells were treated with PD173074 at 6 nM, 25 nM or 100 nM (**A**), or TAE684 at 2 nM, 8 nM or 32 nM (**B**). DMSO diluted in cell growth media served as control. Proliferation curves are shown as mean ± SEM. Two-way ANOVA was used as a statistical test and no significant *p*-values for treatment were observed. (**C**,**D**): Vindelov staining for cell cycle, and (**E**,**F**) Annexin V staining for apoptosis detection was analyzed by flow cytometry. Cells were treated for 48 h with PD173074 (100 nM) or TAE684 (32 nM). Vehicle treatment was DMSO diluted in cell growth media. Experiments were performed in duplicates. Mean ± SEM is shown. Unpaired two-tailed *t*-test was used and significant *p*-values for the comparison of kinase inhibitor treatment versus vehicle control are indicated. (**G**): Endothelial invasion assay. HUVECs form a stable monolayer prior to addition of drug +/− COLO357PL cells. The cell index was measured every 5 min for the first 6 h after addition of cancer cells. The experiment was performed in duplicates. Mean ± SEM is shown. Two-way ANOVA and *p*-values for treatment are indicated.

As a model of human PDAC, we used the human pancreatic cancer cell line COLO357PL, which carries mutant *KRAS* and expresses FGFR and ALK receptors as well as the ALK ligand PTN (Supplementary Figure S1) [13,15,33,34,35,40]. First, we assessed the impact of FGFR and ALK inhibitors on cell proliferation in the presence of different concentrations of an FGFR or ALK kinase inhibitor, PD173074, or TAE684, respectively [41]. Both PD173074 and TAE684 slowed cell growth slightly (Figure 1A,B), suggesting a minor dependence of these cells on autocrine signaling via ligands that activate FGFR and ALK. Vindelov staining for cell cycle analysis as well as an Annexin V apoptosis assay showed that no significant changes in cell cycle distribution and early apoptosis occurred after PD173074 drug treatment, while minimal changes occurred after TAE684 treatment (Figure 1C–F).

We next investigated the effect of FGFR and ALK pathway inhibition on tumor–stroma crosstalk and angiogenesis [16,17,23,42,43]. We measured the ability of cancer cells to disrupt an intact endothelial monolayer, which is monitored by a decline in electrical impedance. This disruption is a step in tumor cell invasion as well as the initial recruitment of endothelial sprouting and can be monitored in real time, as shown previously [44,45]. In the control setting, vehicle treated COLO357PL cancer cells rapidly disrupt the human umbilical vein endothelial cell (HUVEC) monolayer, as indicated by the decline in impedance (cell index). Treatment with PD173074 or TAE684 inhibited the ability of COLO357PL to disrupt HUVEC monolayers in comparison with the vehicle-treated cancer cells (Figure 1G). Neither inhibitor affected the HUVEC monolayer on its own (Figure 1G). These data demonstrate that FGFR and ALK inhibitors disrupt the paracrine crosstalk between cancer cells and the endothelium, and to a greater extent than the inhibition of autocrine-driven cell growth.

### 3.2. FGFR or ALK Inhibitor Treatment Reduces Tumor Cell Proliferation and Angiogenesis In Vivo

**Figure 2:**
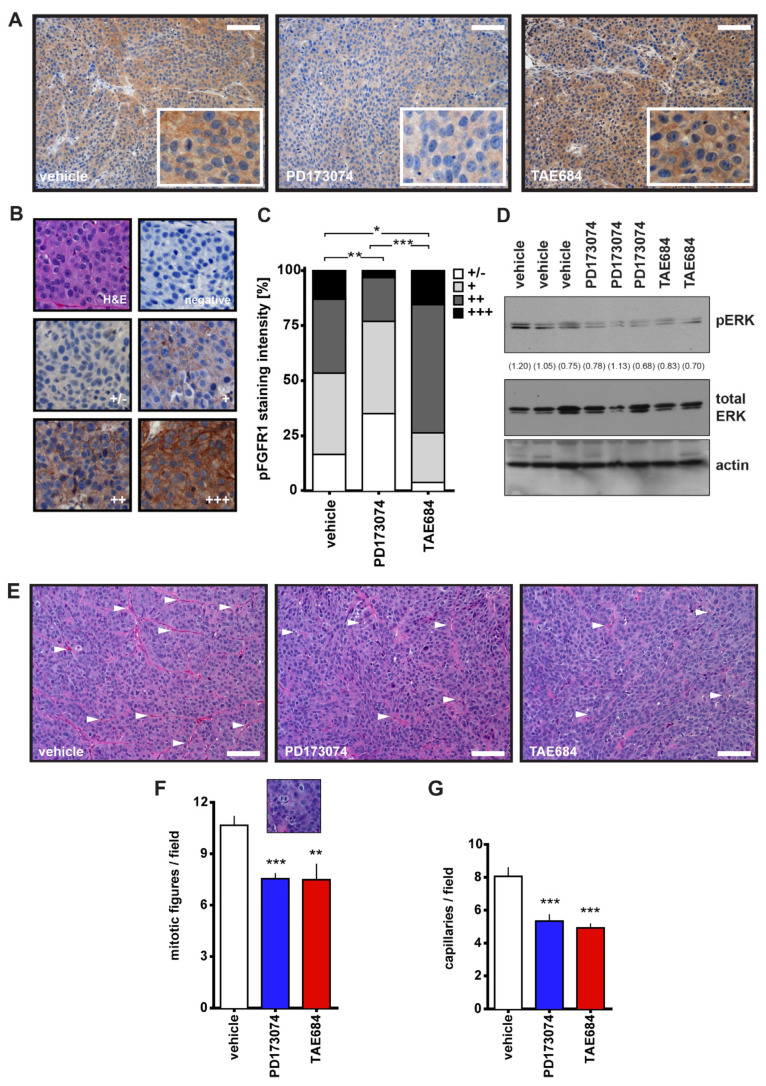
Effect of PD173074 and TAE684 7-day treatment on COLO357PL xenografts. (**A**): phospho-FGFR1 (pFGFR1) staining in representative tumor sections from different treatment groups (vehicle, PD173074 and TAE684), with zoomed inserts showing the localization of the staining. Scale bar, 0.1 mm. (**B**): Representative images of the pFGFR1 staining scale from negative to (+++) staining. (**C**): pFGFR1 staining intensity of the three treatment groups. * *p* < 0.05; ** *p* < 0.01; *** *p* < 0.0001; chi-square test. (**D**): Western blot for phospho- and total ERK of protein lysates from frozen tumor samples. (**E**): Representative H&E-stained tumor sections of the three treatment groups. Scale bar, 0.1 mm. (**F**,**G**): Ten pictures of different fields per tumor sample were analyzed for mitotic figures (**F**) and the number of capillaries (**G**). Mean ± SEM, ** *p* < 0.01; *** *p* ≤ 0.0002; unpaired two-tailed *t*-test. White arrowheads in panel (**E**) indicate capillaries. The insert in panel (**F**) shows an example of a mitotic figure. All the whole western blot figures can be found in the supplementary materials.

To assess the effects of PD173074 and TAE684 on COLO357PL tumors in vivo, we used a xenograft model of subcutaneous tumors in athymic nude mice. After tumors were measurable (~25 mm^2^), mice were treated daily for seven days with either 1 mg/kg of PD173074, 10 mg/kg of TAE684, or vehicle. Tumor and serum samples were collected for further analysis. The activity of PD173074 was reflected by a significant decrease in the intratumoral level of phospho-FGFR1 when compared to the vehicle-treated or TAE684-treated mice (Figure 2A–C). Interestingly, FGFR1 phosphorylation was increased in the tumor tissues after TAE684 treatment, suggesting that the FGFR pathway might compensate for inhibition of ALK signaling (Figure 2A–C). The efficacy of both inhibitors was further demonstrated by analysis of downstream signaling through activation of the MAPK pathway. ERK phosphorylation in the tumor tissue lysates was decreased after treatment with either drug (Figure 2D).

Although overall tumor size after seven days of treatment with FGFR and ALK inhibitors was not affected when compared with vehicle, there was a significant reduction in cancer cell proliferation as measured by the number of mitotic figures (Figure 2E,F) and a reduction in the number of tumor-infiltrating capillaries (Figure 2E,G). Decreased angiogenesis in tumors from PD173074-treated animals was further corroborated by von Willebrand factor (vWF) staining, which marks endothelial cells in larger vessels (Supplementary Figure S2). vWF staining did not differ in tumors from TAE684-treated animals (Supplementary Figure S2). A comparison of the data in Figure 2G and Figure S1 indicates that it is predominantly smaller capillaries that are impacted by the treatment with TAE684.

### 3.3. Effects of FGFR and ALK Inhibitor Treatment on Serum and Tumor miRs

Circulating miRs were recognized as potential cancer biomarkers soon after their detection in the circulation [28,29]. In 2011 we reported distinct changes in serum miR composition upon gemcitabine treatment of control versus cancer-bearing mice [46]. Furthermore, Ohuchida et al. proposed the use of miR expression profiles in tumor tissues as a predictive marker for gemcitabine response after surgical resection of pancreatic cancer [47]. To investigate whether serum miR levels are affected by the seven-day treatment analyzed in Figure 2, we surveyed the expression levels of 352 miRs in the circulation of these mice. Serum samples from mice that belonged to the same experimental group were initially pooled and analyzed by a qPCR expression array. Significantly different miRs were then measured in individual animals. As comparisons, we treated healthy control mice with the inhibitors, followed by analysis of their serum samples.

#### 3.3.1. Host Response to Tumor Burden Indicated by Changes in Serum miRs

**Figure 3:**
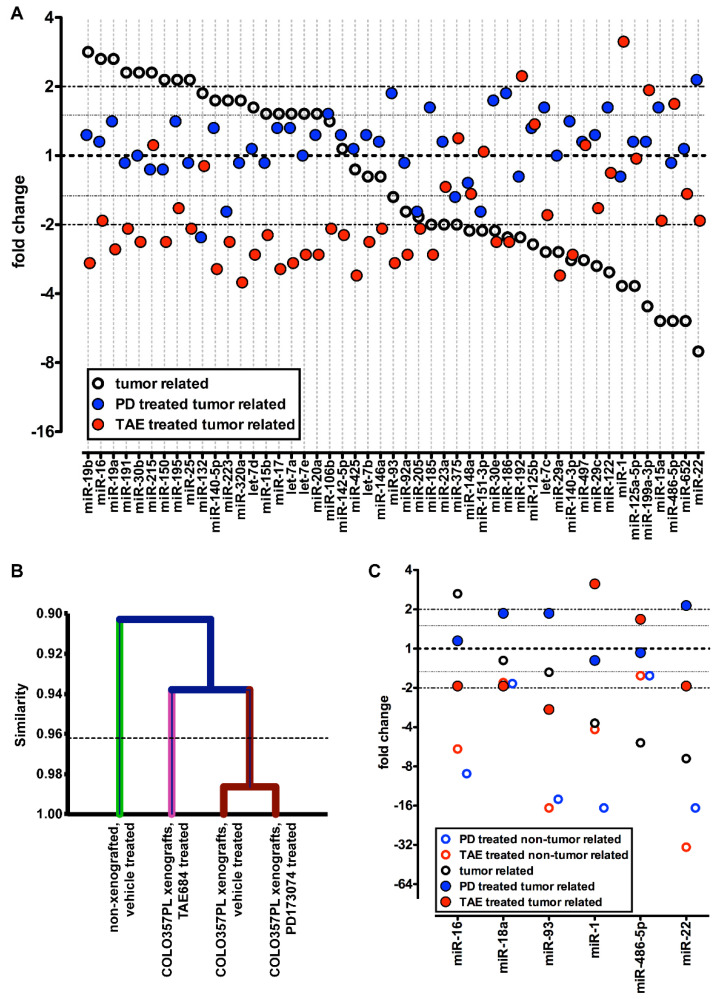
Serum miRs in nude mice with and without tumors and drug treatment. (**A**): Effect of tumor xenografts (black circles); fold changes of serum miRs in mice with COLO357PL tumors relative to controls without tumors. Effect of a 7-day drug treatment on serum miRs in COLO357PL tumor bearing mice (PD173074, blue symbols; TAE684, red symbols); fold changes in serum miRs after treatment relative to control treatment. qPCR was used to analyze 352 miRs, which were normalized for median Ct value. Fold changes are arranged by the expression of tumor-related miRs (black circles). Forty-nine serum miRs with Ct < 30 in every experimental group that were up- or downregulated ≥ two-fold in at least one of the comparisons are included. (**B**): Hierarchical clustering based on serum miR expression. The dotted line indicates *p* < 0.05. (**C**): Effect of PD173074 (blue circles) or TAE684 (red circles) treatment of mice without tumors. Six serum miRs were analyzed for expression in serum samples of nude mice without cancer that underwent short-term treatment. Data were normalized for U6 small nuclear RNA. Data from COLO357PL xenografted mice from panel (**A**) are included for comparison.

To identify serum miRs that are deregulated due to tumor burden (tumor-related miRs), we compared the serum miR levels of vehicle-treated mice with tumors (*n* = 7) to the serum miR levels in healthy mice (*n* = 6). Significantly deregulated miRs are defined as those that are detectable by qPCR (Ct values < 30) and at least two-fold deregulated in tumor-bearing mice relative to healthy mice. Many serum miRs were downregulated in tumor-bearing mice, among which miR-15a, miR-22, miR-128, miR-222, miR-486-5p, miR-574-5p, and miR-652 were decreased more than five-fold (open black circles in Figure 3A, Table 1 and Table 2). In contrast, circulating miR-16, miR-19a, miR-19b, miR-25, miR-30b, miR-150, miR-191, and miR-195 were increased more than two-fold in tumor-bearing mice.

**Figure 4:**
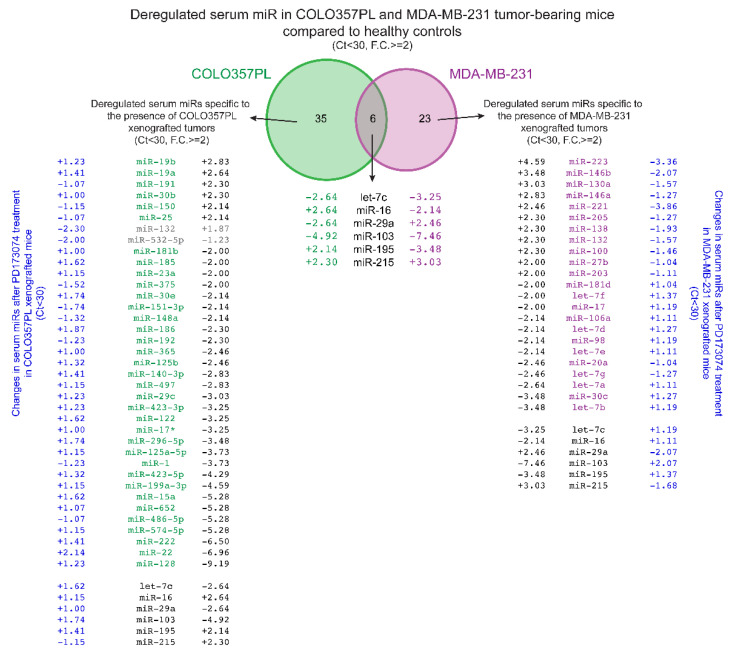
Venn diagram of serum miRs related to the presence of COLO357PL and MDA-MB-231 xenograft tumors, and serum miRs related to the short-term treatment with the FGFR inhibitor, PD173074. The expression levels of 352 serum miRs in COLO357PL (green) and 88 serum miRs in the MDA-MB-231 (magenta) xenograft mouse model were analyzed by qPCR and normalized for respective median Ct value. miRs with Ct < 30 that were changed by ≥2-fold are presented. Additionally, serum miRs changes after short-term treatment with PD173074 (blue) in both xenograft models are presented. Fold changes in serum miR expression after the PD173074 treatment are presented for every tumor-related miR with Ct < 30, as well as those that are ≥2-fold deregulated after PD173074 treatment.

A comparison of tumor-specific circulating miRs with a different xenograft cancer model, namely breast cancer (MDA-MB-231), revealed both cancer type-specific and common cancer-related miR signatures. For example, MDA-MB-231 xenografts are characterized by >3-fold increase in serum miR-130a, miR-146b, miR-215, and miR-223 and >3-fold decrease in serum let-7b, let-7c, miR-30c, miR-103, and miR-195. Interestingly, circulating let-7c and miR-103 are decreased in both breast and pancreatic cancer xenografted animals, while miR-215 is increased in both models. In contrast, miR-16 and miR-195 are increased in serum from animals bearing pancreatic tumors and decreased in response to breast cancer xenografts, while miR-29a is decreased in pancreatic cancer and increased in the breast cancer xenograft model. (Figure 4, Table 1).

#### 3.3.2. Response to Treatment of COLO357PL Tumor-Bearing Mice Indicated by Changes in Serum miRs

Next, we wished to determine whether treatment of COLO357PL tumor-bearing mice was reflected in altered serum miR levels and to identify treatment-related miRs. For this, we compared serum miR levels in kinase inhibitor-treated tumor-bearing mice (*n* = 6 for PD173074; *n* = 6 for TAE684) and vehicle-treated tumor-bearing mice (*n* = 7). Circulating miRs altered after PD173074 treatment include miR-18a, miR-22, miR-93, miR-101, and miR-186, which were increased ~two-fold, while miR-132, miR-203 and miR-342-3p were decreased ~two-fold (Table 2, Figure 3A—blue symbols, Figure 4). Interestingly, the cancer-related decrease in serum miR-22 compared to healthy controls is reversed after PD173074 treatment. miR-22 is associated with senescence in human fibroblast and epithelial cells, and multiple studies have shown that increased miR-22 inhibits cancer cell growth by inducing cell cycle arrest [48,49,50,51]. In contrast, miR-132 was increased 1.9-fold in serum of pancreatic cancer bearing mice compared to healthy controls, and >2-fold decreased after PD173074 treatment. miR-132 is considered to be oncogenic in pancreatic cancer, and has been associated with poor prognosis [52].

A comparison of serum miRs related to the PD173074 treatment of pancreatic cancer-bearing mice with a breast cancer xenograft model (MDA-MB-231) shows >1.5-fold increase in serum miRs with tumor-suppressor properties (miR-103 and miR-122) and a >1.5-fold decrease in oncogenic miR-132, as well as in miR-223. (Figure 4, Table 1 and Table 2). We propose that this particular circulating miR pattern is the PD173074 treatment signature.

TAE684 treatment resulted in a >3-fold increase in serum miR-1 compared to the vehicle-treated mice, whereas miR-17, miR-29a, miR-140-5p, miR-320a, and miR-425 were decreased more than three-fold (Figure 3A—red symbols and Table 2).

#### 3.3.3. In Silico Analysis of Tumor-Related and Treatment-Related Changes of Serum miRs in the COLO357PL Xenograft Mouse Model

Using Ingenuity Pathway Analysis, we found that the deregulated miRs were significantly associated with pathways that are related to cancer and cell cycle (Supplementary Figure S3). Moreover, the tumor-related miRs are linked to pathways involved in gastrointestinal diseases, and both sets of treatment-related miRs are enriched for cell signaling pathways. In addition, the TAE684-related miRs are associated with drug metabolism and small molecule biochemistry.

Hierarchical clustering analysis based on serum miR expression levels showed a separation between the healthy control mice and the tumor-bearing mice. Intriguingly, the vehicle-treated tumor-bearing mice clustered with the PD173074-treated mice, well separated from the TAE684-treated group (Figure 3B). This global analysis, as well as the distinct changes seen for individual miRs, demonstrates that effects of the two kinase inhibitors are biologically distinct.

#### 3.3.4. Host Response to FGFR and ALK Inhibitors Indicated by Changes in Serum miRs

It is likely that treatment-induced changes of serum miRs in tumor-bearing mice include the effects on miRs shed from the tumor as well as other host tissues. To evaluate the impact of drug treatment on host tissues, we treated healthy mice (*n* = 5 per group) for seven days with the FGFR or ALK inhibitor. We then isolated miRs from serum and measured the levels of the six most regulated miRs detected during treatment of tumor-bearing mice. Serum levels of these miRs were reduced in inhibitor-treated healthy mice compared to the treatment-naïve controls (Figure 3C). Taken together, these data show that cancer tissues impact serum miR composition.

#### 3.3.5. Comparison of Circulating and Tumor-Related miRs in COLO357PL Xenograft Mouse Model

We next explored the correlation between changes in miR profiles from tumor tissues and serum from cancer-bearing mice. Tumor miR levels were assessed by qPCR for some of the most deregulated miRs identified in the analysis of serum samples. We anticipated an incomplete correlation, given that circulating miRs are shed from different host tissues. Indeed, certain miRs were not enriched or reduced to the same extent within the tumor as they were in the circulation. For instance, in the vehicle-treated mice the expression levels of miR-126 and miR-192 were higher in the tumors than in serum, whereas miR-486-5p expression was higher in serum than in the tumors (Supplementary Figure S4).

**Figure 5:**
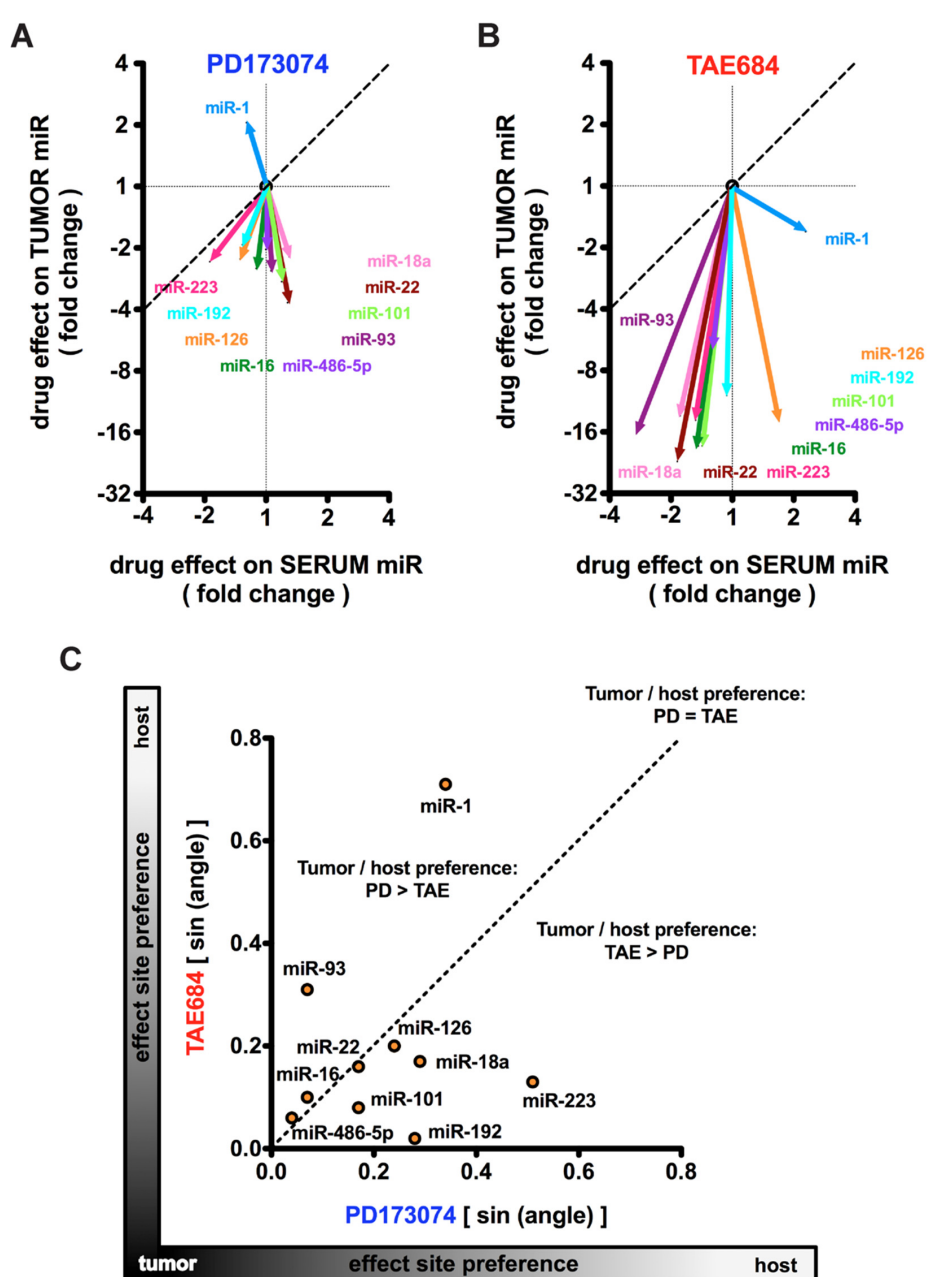
Comparison of serum and tumor miR expression in COLO357PL xenograft mouse model. (**A**,**B**): PD173074 (**A**) or TAE684 (**B**) treatment-induced changes of miRs in serum and tumor samples relative to vehicle treatment. The vectors indicate the fold change of expression in the serum (*x*-axis) and tumor (*y*-axis) samples harvested at the same time. qPCR for miRs in serum samples were run in duplicates on previously pooled serum samples of mice that belonged to the same treatment group (*n* = 6 and 7 serum samples per treatment group); tumor samples were analyzed separately for miR expression (*n* = 7 and 8 tumors per treatment group). The dotted line indicates identical changes in tumor and serum. (**C**): Tumor or host (serum) preferential effect site for PD173074 versus TAE684 treatment. The sine of the direction of each miR change vector in panels (**A**,**B**) are plotted. The dotted line indicates identical preference of TAE and PD for the respective miR.

To determine which miRs reflect a tumor-specific response to treatment (either PD173074 or TAE684) and those which represent a host-targeting effect, we compared the miR profiles in the serum and tumors from kinase inhibitor-treated mice with COLO357PL xenograft tumors (Figure 5A,B). The treatment effects of PD173074 and TAE684 are depicted as fold changes relative to the vehicle controls. We used vectors to visualize the relative change of each miR in the serum as well as the tumor. The angle of the respective vectors relative to the vertical axis reflects the extent to which drug treatment impacted the tumor-derived miR. A 45° angle between a vector and the vertical axis implies identical changes of a given miR in the tumor tissues and the serum. A smaller angle (<45°) implies a larger contribution of the changes in the particular miR from the tumor tissue and a smaller contribution from host tissues. Conversely, an angle of >45° for the respective miR, primarily reflects a host tissue response to treatment.

For the selected top ten deregulated miRs identified in the analysis of serum samples, the angles between the vector and the vertical identity line range from 2° to 31° for PD173074 treatment and from 1° to 45° for TAE684 treatment. Thus, the dominant direction of the vectors indicates that changes in the serum miRs are mostly due to the tumor response to treatment. In addition, TAE684 treatment caused much greater overall changes in miR levels than PD173074 (Figure 4, Table 2).

To further compare the miR profiles between PD173074- and TAE684-treated mice, we plotted the sine values of the angles between the vectors and the vertical identity line (Figure 5C). From this analysis, one can deduce that miR-1 and miR-93 represent tumor-specific effects of PD173074, whereas miR-192 and miR-223 represent tumor-specific effects of TAE684. In addition, miR-486-5p, miR-16, miR-22, and miR-126 reflect tumor response to both treatments. Interestingly, our data indicate that the magnitude of the tumor response to treatment was significantly greater than the host response, suggesting a larger contribution of tumor tissues to the miRs shed into the circulation upon treatment. This implies that the tumors are more susceptible to the inhibitors than healthy tissues.

### 3.4. Effect of Three-Week Treatment with PD173074 or TAE684 on COLO357PL Tumor Xenografts

**Figure 6:**
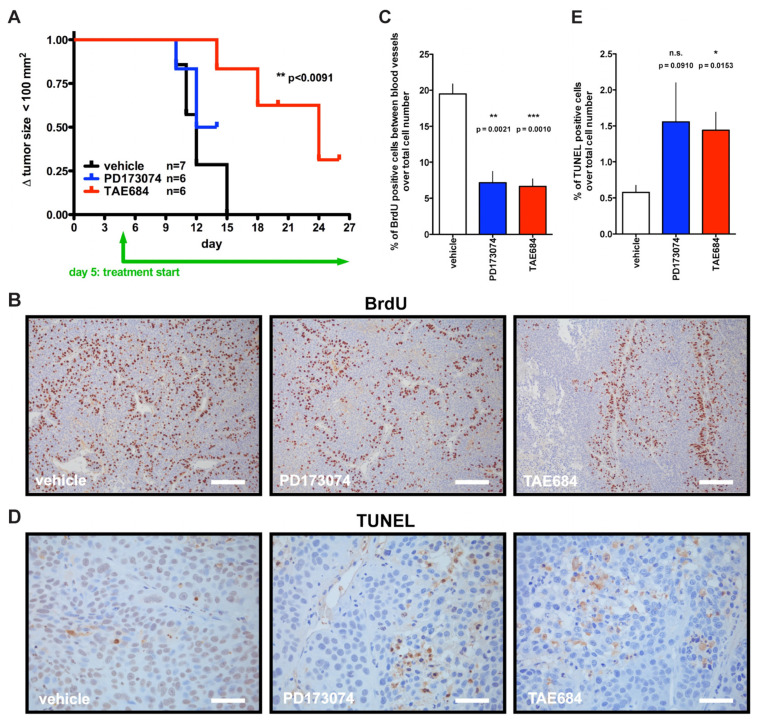
Effect of PD173074 and TAE684 treatment on COLO357PL tumor xenografts. (**A**): Kaplan–Meier plot of tumor growth >100 mm^2^ during the three-week treatment period. TAE684 vs. control; *p*-value by Gehan–Breslow–Wilcoxon test is indicated. PD173074 *p* > 0.05 vs. control. (**B**,**C**): BrdU staining for proliferating cells. Representative images of immunostaining are shown. Scale bar, 0.2 mm (**B**). Proliferating cells > 3 cell layers distant from blood vessels were quantified (**C**). A minimum of 22 images per group (total of 71) were analyzed. (**D**,**E**): TUNEL staining for apoptotic cells. Representative pictures are shown. Scale bar, 0.05 mm (**D**). Panels (**B**–**E**): Vehicle treated mice, *n* = 4; drug treatment groups, *n* = 3 each; mean ± SEM; *p*-values from unpaired two-tailed *t*-test.

To determine which inhibitor is better at delaying and/or preventing tumor progression, we performed a longer experiment with daily treatment of mice with PD173074 or TAE684 initiated after establishment of subcutaneous COLO357PL tumors (≥25 mm^2^) and continued until at least one of the two subcutaneous tumors per mouse reached ≥125 mm^2^ as defined by the animal protocol endpoint. TAE684-treated mice had significantly delayed tumor growth compared to the control group (Figure 6A). In contrast, there was no difference between controls and PD173074-treated mice (Figure 6A).

Tumor cell proliferation as assessed by BrdU staining showed a similar staining rate for cells located close to blood vessels (within three cell layers) for all treatment groups. However, the control group showed BrdU-positive cells distant from blood vessels (beyond three cell layers), and the quantitation revealed more than a three-fold increase in actively dividing cells in the areas between blood vessels in the control group compared to the treated groups (Figure 6B,C). This indicates that both treatments impacted the angiogenic supply of the growing tumors, and complements the results from the short-term treatment in which we counted fewer actively dividing cells in the tumors of treated mice than in the controls. In addition, TUNEL staining revealed three-fold more apoptotic cancer cells in tumors from the TAE684-treated mice than in the vehicle-treated group (Figure 6D,E). TUNEL staining trended higher in the PD173074-treated tumors relative to control as well, though this increase did not reach statistical significance.

Taken together, the distinct outcome from this longer-term study based on the kinase inhibitor used is consistent with the distinct response of circulating miRs derived from the seven-day treatment (Figure 3A,B and Figure 5A,B, Table 2). Given that the changes in serum miRs occurred before observable changes in tumor size, this suggests that a therapeutic response can become apparent from an early analysis of changes in circulating miRs.

## 4. Discussion

In the present study, we evaluated the utility of changes in circulating miR signatures as readouts of drug efficacy. We first needed to establish a ‘diagnostic’ set of circulating miRs associated with the presence of tumor xenografts. From our study of serum miRs in the xenograft models of pancreatic (COLO357PL) and breast (MDA-MB-231) cancer, we found two distinct miR profiles with an overlap in six serum miRs, three of which (let-7c, miR-103 and miR-215) changed in the same direction in both cancer types (Figure 4, Table 1). Interestingly, loss of let-7 family members is associated with poorly differentiated aggressive cancers, including pancreatic and breast cancer [53,54,55]. The serum miR signature showed a marked ≥3-fold increase in miR-130a, miR-146b, miR-215, and miR-223 and a >3-fold decrease of miR-30c, miR-103, miR-195, let-7b, and let-7c in MDA-MB-231 xenografted mice when compared to healthy controls, which differs from the pancreatic cancer-related circulating miR pattern. The set specific to the pancreatic cancer described here (>2-fold increased circulating miR-16, miR-19a, miR-19b, miR-25, miR-30b, miR-150, miR-191, and miR-195 and >5-fold decreased miR-15a, miR-22, miR-128, miR-222, miR-486-5p, miR-574-5p, and miR-652) matches partly, although not completely, with previously published studies [56,57,58]. Potential reasons for this discrepancy might be due to the actual analysis of samples from humans compared to animal models that either use human xenografts in immune-compromised mice or immune competent syngeneic mouse models. In addition, the selection criteria for miRs with significant changes may vary among different platforms used for miR detection. Here, we used a cutoff of ≥2-fold deregulation in miR expression with a qPCR readout of Ct < 30 cycles. Furthermore, miR expression normalization methods vary between studies. In the present analysis, we normalized data to the median level of the panel of 352 miRs analyzed, not to the level of a housekeeping gene such as RNU6. Therefore, different models, selection criteria, normalization methods, and miR panels available may contribute to discrepancies in ‘diagnostic’ sets of miRs in different studies.

Furthermore, we identified two distinct sets of serum miRs that showed a differential change in response to either an FGFR or an ALK kinase inhibitor. The ALK kinase inhibitor had a greater overall impact on circulating miR expression changes, which is reflected in a more pronounced long-term treatment effect in vivo. Furthermore, our analysis allowed us to distinguish between the tumor and host effect of the drug treatment. Although both inhibitors had similar initial effects on tumor angiogenesis and mitosis as well as MAPK signaling, the effects of the two drugs impacted distinct sets of serum miRs. We propose that the treatment-specific circulating miR signature reflects subtle drug-related changes in components of the tumor microenvironment and/or the host that escape detection by standard histological and signaling pathway analyses (Figure 2). Hence, we suggest that changes in serum miRs upon treatment may precede histological changes.

We found serum miR-103 and miR-122 with tumor suppressor properties that are more than 1.5-fold upregulated, and oncogenic miR-132 and miR-223 more than two-fold downregulated after PD173074 treatment of the xenograft models of pancreatic and breast cancer (Table 1). Notably, miR-103 was decreased in mice with either cancer type relative to the healthy controls, and PD173074 treatment increased its levels. miR-103 is increased under hypoxia and promotes apoptosis of endothelial cells through direct targeting of the anti-apoptotic protein Bcl-XL [59]. Given that the number of capillaries in tumor tissues was reduced in the pancreatic cancer model after PD173074 treatment, it is possible that a hypoxic state could lead to the increase of miR-103. Furthermore, miR-122 is considered a tumor-suppressing miR that is decreased in hepatocellular carcinoma and metastatic disease [60,61,62]. Consistently, increased miR-122 is linked to enhanced apoptosis of hepatoma cells [63,64]. Here, circulating miR-103 and miR-122 are increased after short-term PD173074 treatment, suggesting that this FGFR inhibitor may mediate their tumor-suppressing activity. In contrast, serum miR-132 and miR-223 were >1.7-fold upregulated in pancreatic and breast cancer xenograft models in comparison to healthy control, followed by their >1.5-fold downregulation after PD173074 treatment. Importantly, it has been shown that miR-132 was increased in patient PDAC tissues compared to normal pancreas and adjacent benign pancreas [65], and its expression was associated with poor prognosis in PDAC [52]. Similarly, miR-223 was found to be increased in pancreatic cancer patients in comparison to healthy controls, and was decreased postoperatively [66]. Additionally, it has been shown that miR-223 regulates pancreatic cancer cell proliferation and invasion in vitro and in vivo [67]. However, changes observed for miR-223 may be impacted by the high expression of this miR in myeloid cells [68].

Although the two tumor suppressor miRs described above were increased upon short-term PD173074 treatment in both xenograft cancer models, we did not observe a survival benefit after the prolonged treatment of pancreatic cancer-bearing mice. Perhaps the changes in miRs after PD173074 treatment at the dose of 1 mg/kg are not sufficient, as suggested by the strikingly larger changes in serum miRs upon TAE684 treatment. Specifically, serum miR-1 is upregulated in tumor-bearing mice treated with the ALK inhibitor, while the same miR is unaffected in the serum of mice treated with the FGFR inhibitor. Interestingly, increased circulatory miR-1 of patients with non-small-cell lung cancer and hepatocellular carcinoma predicted longer overall and progression-free survival [69,70,71]. Importantly, another study showed a decrease in miR-1 expression in PDAC patient sera or tumors in comparison to healthy controls, and higher miR-1 expression was associated with longer survival in PDAC patients [72]. Here, we observed a favorable outcome after prolonged treatment with TAE684, which was associated with a three-fold increase in serum miR-1 (Table 2). However, we do not advocate for the use of a single miR as a marker of therapy response. Rather, we suggest using a pattern of several miRs for the prediction of a treatment response. However, more preclinical and clinical studies will be needed to translate the findings robustly into clinical utility. Indeed, we have previously demonstrated that serum miRs can be used for following response to gemcitabine in genetically engineered mouse model of PDAC [46], as well as for predicting PDAC recurrence after tumor resection in pancreatic cancer patients, which correlated with the genetically engineered mouse model of PDAC [73].

## 5. Conclusions

In conclusion, our data show that different targeted therapies can result in unique circulating miR profiles even when initial treatment responses are indistinguishable by typical measures such as histology and signal transduction. The present study can be used as a proof-of-principle for predicting treatment responses based on the assessment of serum miR patterns shortly after treatment initiation, and demonstrates that treatment-induced changes in circulating miRs could provide a signature indicative of efficacy.

## Figures and Tables

**Table 1:** Serum miRs related to the presence of COLO357PL and MDA-MB-231 xenograft tumors and serum miRs related to the short-term treatment with the FGFR inhibitor, PD173074. The expression levels of 352 serum miRs in COLO357PL and 88 serum miRs in the MDA-MB-231 xenograft mouse model were analyzed by qPCR and normalized for the respective median Ct value. miRs with Ct < 30 that were changed by ≥2-fold in at least one comparison are presented. Additionally, serum miRs changes after short-term treatment with PD173074 in both xenograft models are presented. Fold changes in serum miR expression after PD173074 treatment are presented for every tumor-related miR with Ct < 30, as well as those ≥2-fold deregulated after PD173074 treatment.

	COLO357PL		MDA-MB-231	
	Drug + Xenograft	Xenograft	Xenograft	Drug + Xenograft
	PD173074 Treatment of COLO357PL Xenografted Mice	+COLO357PL	+MDA-MB-231	PD173074 Treatment of MDA-MB-231 Xenografted Mice
miR	Fold Change 2^(−^^ΔΔ^^Ct)^	Fold Change 2^(−^^ΔΔ^^Ct)^	Fold Change 2^(−^^ΔΔ^^Ct)^	Fold Change 2^(−^^ΔΔ^^Ct)^
let-7a	1.32	1.52	−2.64	1.11
let-7b	1.23	−1.23	−3.48	1.19
let-7c	1.62	−2.64	−3.25	1.19
let-7d	1.07	1.62	−2.14	1.27
let-7e	1.00	1.52	−2.14	1.11
let-7f	−1.23 #	4.00 #	−2.00	1.37
let-7g	1.52	−1.32	−2.46	−1.27
miR-1	−1.23	−3.73	1.23	−1.19
miR-15a	1.62	−5.28	−1.74	−1.04
miR-16	1.15	2.64	−2.14	1.11
miR-17	1.32	1.52	−2.00	1.19
miR-17-3p	1.00	−3.25	N/A	N/A
miR-19a	1.41	2.64	−1.23	−1.68
miR-19b	1.23	2.83	N/A	N/A
miR-20a	1.23	1.52	−2.46	−1.04
miR-22	2.14	−6.96	N/A	N/A
miR-23a	1.15	−2.00	N/A	N/A
miR-25	−1.07	2.14	−1825676.85 #	1085553.95 #
miR-27b	1.15	1.32	2.00	−1.04
miR-29a	1.00	−2.64	2.46	−2.07
miR-29c	1.23	−3.03	N/A	N/A
miR-30b	1.00	2.30	N/A	N/A
miR-30c	1.74	−1.41	−3.48	1.27
miR-30e	1.74	−2.14	N/A	N/A
miR-98	−1.62 #	−1.23 #	−2.14	1.19
miR-100	1.07	1.32	2.30	−1.46
miR-103	1.74	−4.92	−7.46	2.07
miR-106a	N/A	N/A	−2.14	1.11
miR-122	1.62	−3.25	1.07	1.93
miR-125a-5p	1.15	−3.73	−1.23	1.27
miR-125b	1.32	−2.46	N/A	N/A
miR-128	1.23	−9.19	1.32	−1.27
miR-130a	1.15	2.30 #	3.03	−1.57
miR-132	−2.30	1.87	2.30	−1.57
miR-138	−1.41 #	−4.29 #	2.30	−1.93
miR-140-3p	1.41	−2.83	N/A	N/A
miR-146a	1.15	−1.23	2.83	−1.27
miR-146b	1.52	1.62	3.48	−2.07
miR-148a	−1.32	−2.14	1.62	−1.68
miR-150	−1.15	2.14	1.15	1.57
miR-151-3p	−1.74	−2.14	N/A	N/A
miR-181b	1.00	−2.00	−1.74	1.27
miR-181d	1.32 #	−2.46 #	−2.00	1.04
miR-185	1.62	−2.00	N/A	N/A
miR-186	1.87	−2.30	N/A	N/A
miR-191	−1.07	2.30	−1.52	1.37
miR-192	−1.23	−2.30	N/A	N/A
miR-195	1.41	2.14	−3.48	1.37
miR-199a-3p	1.15	−4.59	N/A	N/A
miR-203	−1.87	1.00	2.00	−1.11
miR-205	−1.74	−1.87	2.30	−1.27
miR-215	−1.15	2.30	3.03	−1.68
miR-221	N/A	N/A	2.46	−3.86
miR-222	1.41	−6.50	−1.32	−1.37
miR-223	−1.74	1.74	4.59	−3.36
miR-296-5p	1.74	−3.48	N/A	N/A
miR-365	1.00	−2.46	N/A	N/A
miR-375	−1.52	−2.00	N/A	N/A
miR-423-3p	1.23	−3.25	N/A	N/A
miR-423-5p	1.32	−4.29	N/A	N/A
miR-486-5p	−1.07	−5.28	N/A	N/A
miR-497	1.15	−2.83	N/A	N/A
miR-532-5p	−2.00	−1.23	N/A	N/A
miR-574-5p	1.15	−5.28	N/A	N/A
miR-652	1.07	−5.28	N/A	N/A

#—Ct value > 30; N/A—not applicable (the particular miR was not available in the array panel).

**Table 2:** Serum miRs related to the presence of COLO357PL xenograft tumors, and serum miRs related to short-term treatment with the FGFR inhibitor PD173074 or the ALK inhibitor TAE684. The expression levels of 352 serum miRs in a COLO357PL xenograft mouse model were analyzed by qPCR and normalized for respective median Ct value. miRs with Ct < 30 in at least one comparison are presented.

	Xenograft	Drug + Xenograft	
	+COLO357PL (Vehicle Control)	PD173074 Treatment of COLO357PL Xenografted Mice	TAE684 Treatment of COLO357PL Xenografted Mice
miR	Fold Change 2^(−^^ΔΔCt)^	Fold Change 2^(−^^ΔΔCt)^	Fold Change 2^(−^^ΔΔCt)^
let-7a	1.52	1.32	−2.93
let-7b	−1.23	1.23	−2.38
let-7c	−2.64	1.62	−1.80
let-7d	1.62	1.07	−2.73
let-7e	1.52	1.00	−2.73
let-7g ‡	−1.32	1.52	−1.80
let-7i ‡	−1.07	1.41	−1.93
miR-1	−3.73	−1.23	3.14
miR-7 ##	1.74	1.23	−2.22 #
miR-15a	−5.28	1.62	−1.93
miR-15b	1.52	−1.07	−2.22
miR-16	2.64	1.15	−1.93
miR-17	1.52	1.32	−3.14
miR-17-3p ##	−3.25	1.00	−1.11 #
miR-18a ‡	−1.23	1.87	−1.93
miR-19a	2.64	1.41	−2.55
miR-19b	2.83	1.23	−2.93
miR-20a	1.52	1.23	−2.73
miR-20b ‡	1.74	−1.15	−1.37
miR-21 ‡	1.41	1.23	−1.68
miR-22	−6.96	2.14	−1.93
miR-23a	−2.00	1.15	−1.37
miR-23b ‡	−1.32	1.00	−1.57
miR-24 ‡	1.07	1.15	−1.93
miR-25	2.14	−1.07	−2.07
miR-26a ‡	−1.41	1.07	−1.57
miR-26b ‡	1.15	1.32	−1.57
miR-27a ‡	1.07	−1.15	−1.68
miR-27b ‡	1.32	1.15	−1.19
miR-29a	−2.64	1.00	−3.36
miR-29c	−3.03	1.23	−1.68
miR-30a ‡	1.32	1.23	−1.93
miR-30b	2.30	1.00	−2.38
miR-30c ‡	−1.41	1.74	−1.27
miR-30d ‡	1.07	1.41	−1.68
miR-30e	−2.14	1.74	−2.38
miR-92a	−1.74	−1.07	−2.73
miR-93	−1.52	1.87	−2.93
miR-99a ‡	−1.87	1.32	−1.19
miR-100 ‡	1.32	1.07	−1.46
miR-101 ‡	1.52	1.87	−1.57
miR-103 ##	−4.92	1.74	−2.38 #
miR-106b	1.41	1.52	−2.07
miR-122	−3.25	1.62	−1.19
miR-125a-5p	−3.73	1.15	−1.04
miR-125b	−2.46	1.32	1.37
miR-126 ‡	1.00	1.23	1.27
miR-128 ##	−9.19	1.23	−2.93 #
miR-130a ##	2.30 #	1.15	−3.86 #
miR-130b ##	1.87	−1.32	−1.80 #
miR-132	1.87	−2.30	−1.11
miR-139-5p ‡	−1.87	−1.74	1.57
miR-140-3p	−2.83	1.41	−2.73
miR-140-5p	1.74	1.32	−3.14
miR-142-5p	1.07	1.23	−2.22
miR-146a	−1.23	1.15	−2.07
miR-146b-5p ‡	1.62	1.52	−1.46
miR-148a	−2.14	−1.32	−1.46
miR-148b ‡	−1.74	−1.07	−1.04
miR-150	2.14	−1.15	−2.38
miR-151-3p	−2.14	−1.74	1.04
miR-151-5p ‡	1.00	1.15	−1.80
miR-152 ‡	−1.41	−1.74	−1.04
miR-181b ##	−2.00	1.00	−1.57 #
miR-185	−2.00	1.62	−2.73
miR-186	−2.30	1.87	−2.38
miR-191	2.30	−1.07	−2.07
miR-192	−2.30	−1.23	2.22
miR-193b ##	1.07	−1.23	−1.27 #
miR-194 ‡	1.15	1.07	−1.46
miR-195	2.14	1.41	−1.68
miR-199a-3p	−4.59	1.15	1.93
miR-200c ‡	1.15	1.07	−1.80
miR-202 ##	5.66 #	−1.62	1.11
miR-203 ‡	1.00	−1.87	1.11
miR-205	−1.87	−1.74	−2.07
miR-214 ‡	−1.23	−1.32	1.19
miR-215	2.30	−1.15	1.11
miR-222 ##	−6.50	1.41	−2.22 #
miR-223	1.74	−1.74	−2.38
miR-296-5p ##	−3.48	1.74	−3.36 #
miR-301a ‡	1.32	−1.41	−1.37
miR-301b ‡	−1.41	1.07	1.46
miR-320a	1.74	−1.07	−3.61
miR-330-3p ##	1.23	−1.07	−2.73 #
miR-340 ‡	−1.52	1.00	1.46
miR-342-3p ‡	1.52	−1.87	−1.37
miR-361-3p ##	1.07	1.62	−2.38 #
miR-365 ##	−2.46	1.00	−1060.11 #
miR-374b ##	1.15	1.62	−1.27 #
miR-375	−2.00	−1.52	1.19
miR-378 ‡	1.32	−1.32	−1.80
miR-421 ‡	1.87	−1.41	−1.19
miR-422a ‡	1.32	1.23	1.04
miR-423-3p ##	−3.25	1.23	−2.38 #
miR-423-5p ##	−4.29	1.32	−4.14 #
miR-424 ‡	−1.15	1.00	−1.80
miR-425	−1.15	1.07	−3.36
miR-450a ##	3.73 #	1.23	−1.68 #
miR-484 ‡	−1.62	−1.41	−1.93
miR-486-5p	−5.28	−1.07	1.68
miR-497	−2.83	1.15	1.11
miR-532-3p ‡	1.32	1.32	−1.93
miR-532-5p ##	−1.23	−2.00	−1.93 #
miR-574-5p ##	−5.28	1.15	−1.80 #
miR-638 ##	2.46 #	−1.32	−1.46 #
miR-652	−5.28	1.07	−1.46
miR-744 ‡	1.41	1.15	−1.27

#—Ct value > 30; ##—miRs with Ct value > 30; ‡—miRs with fold change <2 in all three comparisons.

## Data Availability

The data presented in this study are available on request from the corresponding author.

## Supplementary Materials

The following are available online at https://www.mdpi.com/article/10.3390/cancers14061517/s1, Figure S1: In silico analysis of gene expression in human tissues and cell lines, Figure S2: Immunohistochemistry for von Willebrand factor (vWF), Figure S3: In silico analysis of serum miR expression, Figure S4: Comparison of serum and tumor miR expression levels in mice with xenograft tumors. All the whole western blot figures can be found in the supplementary materials.
