# Supplementary material for: Distinct Response of Circulating microRNAs to the Treatment of Pancreatic Cancer Xenografts with FGFR and ALK Kinase Inhibitors
Source: Cancers (Basel). 2022 Mar 16;14(6):1517. doi: 10.3390/cancers14061517 (PMC8945909; doi:10.3390/cancers14061517)
Supplement: Supplementary file 1 [file cancers-14-01517-s001.zip › Peran et al Supplementary Materials-IPedits.pdf]

cancers

MDPI

Supplementary Materials

Distinct Response of Circulating microRNAs to the Treatment of Pancreatic Cancer Xenografts with FGFR and ALK Kinase Inhibitors

Ivana Peran, Eveline E. Vietsch, Gai Yan, Anna T. Riegel and Anton Wellstein *

Georgetown-Lombardi Comprehensive Cancer Center, Department of Oncology, Georgetown University Medical Center, Washington, DC 20057, USA; ip62@georgetown.edu (I.P.); e.vietsch@erasmusmc.nl (E.E.V.); gy63@georgetown.edu (G.Y.); ariege01@georgetown.edu (A.T.R.)
* Correspondence: wellstea@georgetown.edu

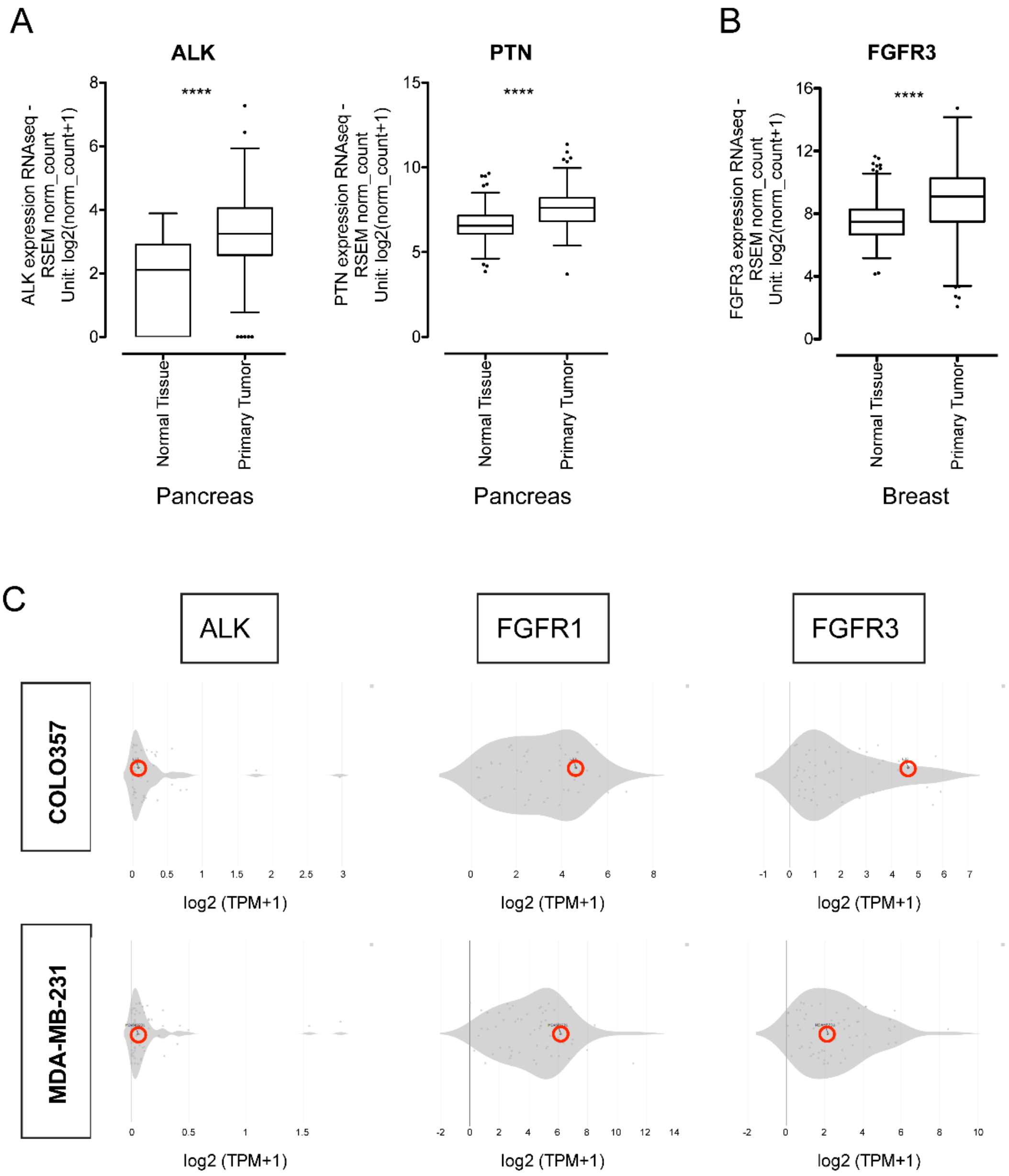

Supplementary Figure S1. *In silico* analysis of gene expression in human tissues and cell lines. **(A)** *ALK* and *PTN* mRNA expression in normal pancreas versus primary pancreatic cancer tissue; **(B)** *FGFR3* mRNA expression in normal breast versus primary breast cancer tissue. Publicly available TCGA and GTEX databases were used through Xenabrowser (https://xenabrowser.net/ by University of California Santa Cruz, CA, USA; last accessed on January 21st, 2021) to extract data. ****p-value < 0.0001, by unpaired two-tailed t-test. **(C)** *ALK*, *FGFR1* and *FGFR3* mRNA expression across more than 1000 cell lines available through the Cancer Cell Line Encyclopedia (CCLE) by the Broad Institute, Cambridge, MA, USA (https://sites.broadinstitute.org/ccle/ last accessed on January 21st, 2021). COLO357 and MDA-MB-231 cell lines are labeled with red circle.

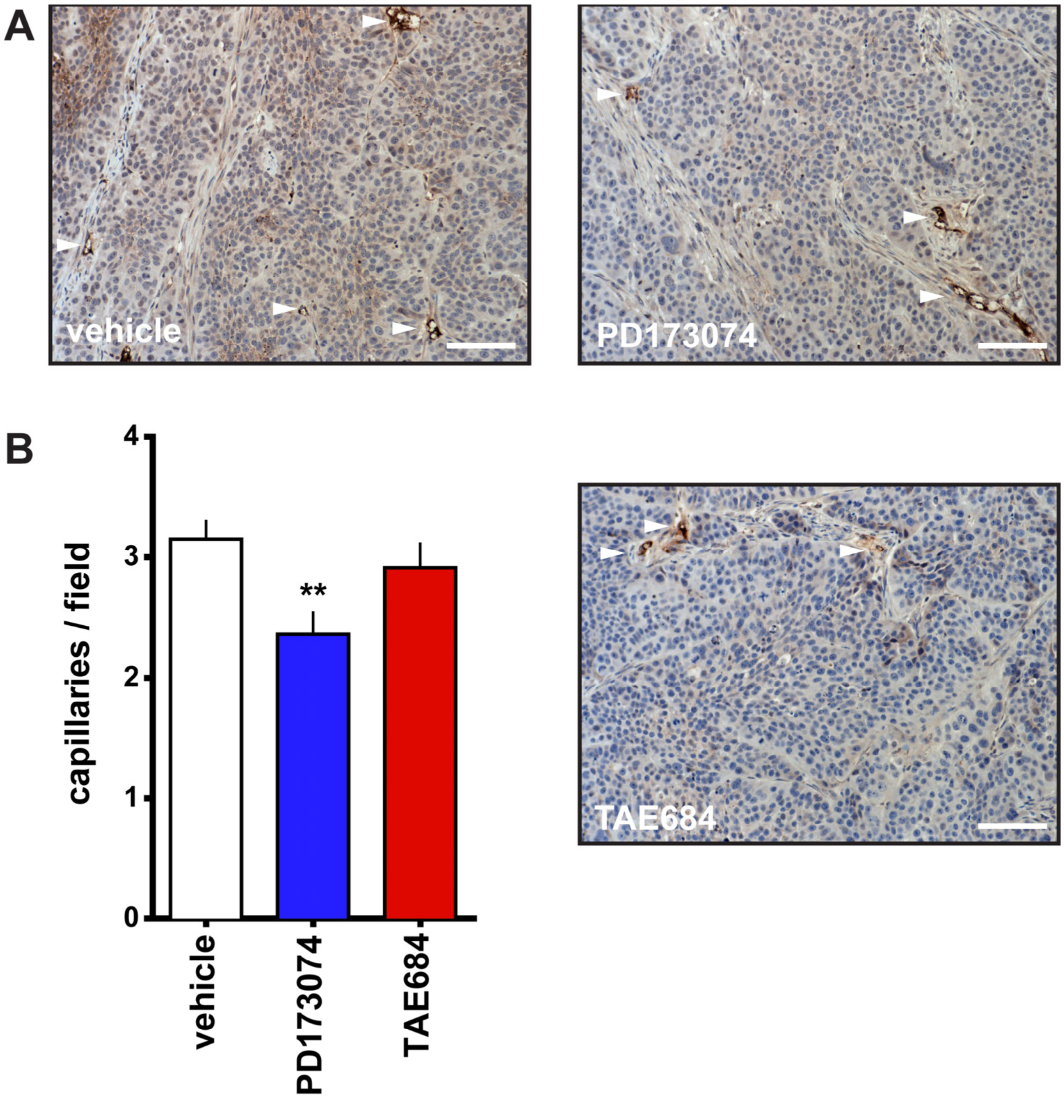

Supplementary Figure S2. Immunohistochemistry for von Willebrand factor (vWF). Ten pictures of different fields per tumor sample were taken for analysis. **(A):** Representative images of tumor samples from the vehicle, PD173074 and TAE684 treatment group are shown. Size bar, 0.1 mm. **(B):** The number of capillaries was assessed after staining for vWF. ** p-value < 0.01 by unpaired two-tailed t-test.

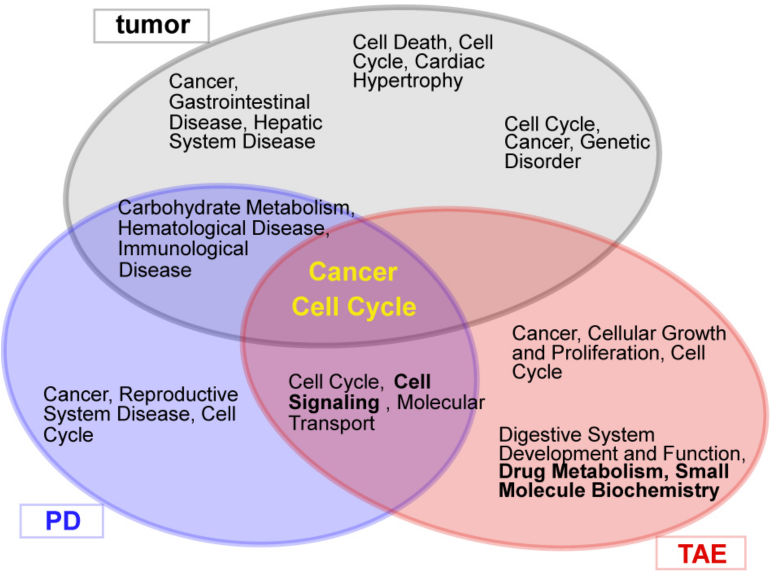

Supplementary Figure S3. *In silico* analysis of serum miR expression. Ingenuity Pathway Analysis (Ingenuity® Systems, Redwood City, CA, USA) of serum miRs related to: Tumor presence, treatment with PD173074 or with TAE684.

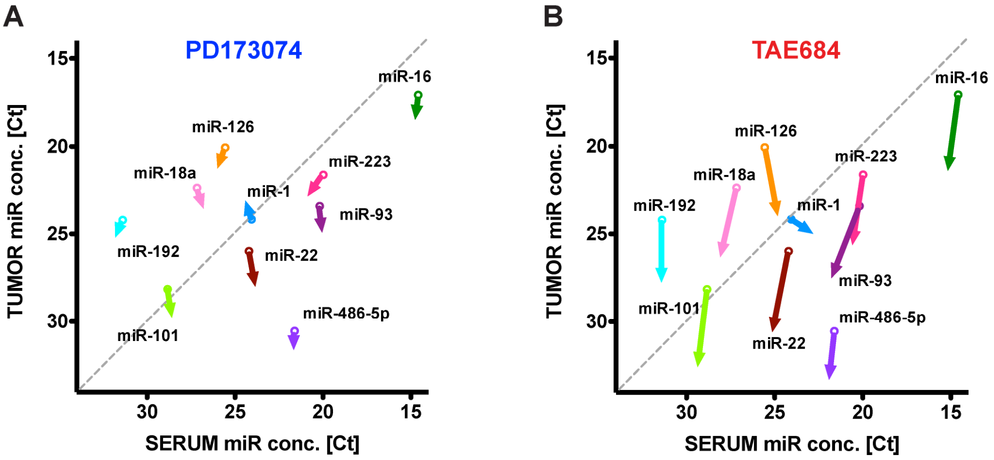

Supplementary Figure S4. Comparison of serum and tumor miR expression levels in mice with xenograft tumors. **(A, B):** PD173074 (A) and TAE684 (B) treatment changes of miR expression levels in serum and tumor samples are compared to vehicle treated mice. Baseline expression level of each miR in vehicle treated mice (open circle) and change in expression due to drug treatment (arrow head). The direction of the vectors indicates whether changes in miR expression due to treatment are preferentially in serum or tumor samples. The lengths of the vectors indicate the change in expression due to treatment. Mean Ct values are shown. SEM for serum samples was <0.25 Ct units, for tumor samples 0.24 to 2.13. qPCRs for miRs in serum samples were run in duplicates on pooled serum samples (n = 6 to 7 mice per treatment group). Tumors from each treatment group (n = 7 to 8 tumors per group) were analyzed separately.
