# Supplementary material for: Distinct Response of Circulating microRNAs to the Treatment of Pancreatic Cancer Xenografts with FGFR and ALK Kinase Inhibitors
Source: Cancers (Basel). 2022 Mar 16;14(6):1517. doi: 10.3390/cancers14061517 (PMC8945909; doi:10.3390/cancers14061517)

Full Western blot scans correspond to the Figure 2D. Western blot for phospho-ERK (pERK) and total ERK of protein lysates from frozen tumor samples.
PD denotes lanes loaded with protein lysates from COLO357PL xenografted mice treated with PD173074, while TAE denotes lanes loaded with protein lysates from COLO357PL xenografted mice treated with TAE684.
(Please note that in the Figure 2D, in all cases, the positive control lane (+ Control) was omitted, and lanes were inverted to show from left to right: 3 vehicle lanes, followed by 3 PD-treated lanes, and finally 2 ALK-treated lanes.)

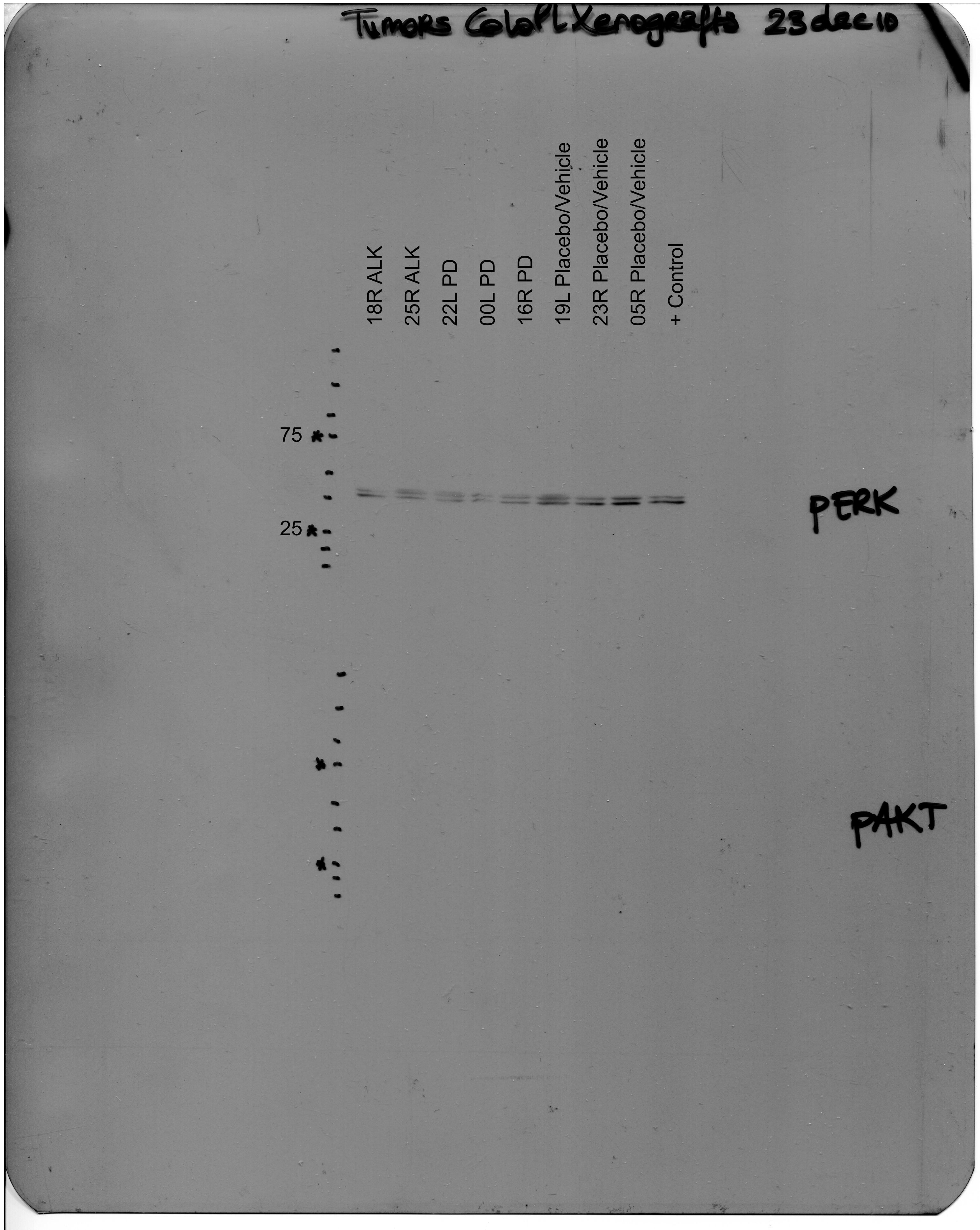

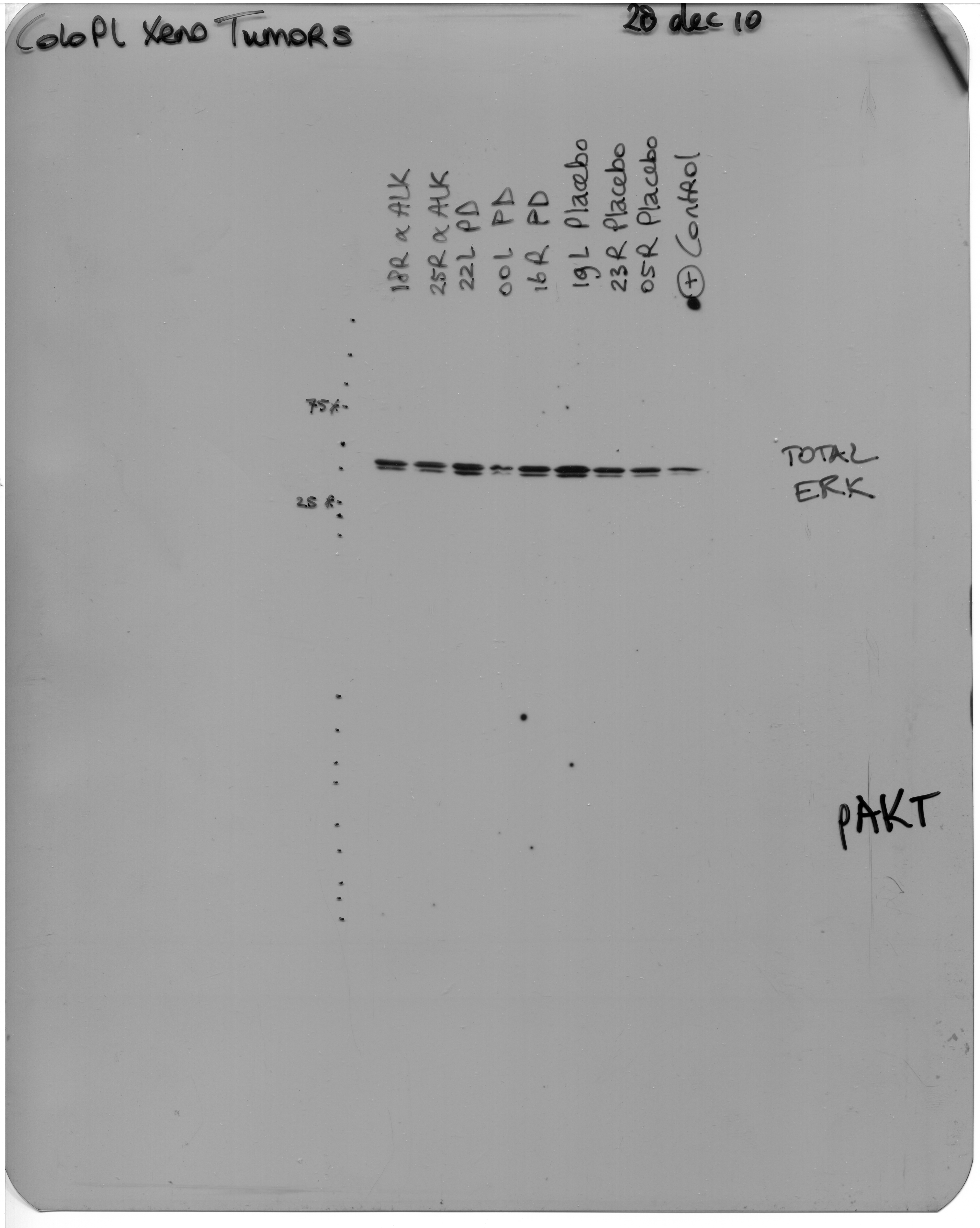

Supplement: Supplementary file 1 [file cancers-14-01517-s001.zip › western.pdf]
